# Supplementary figures and images for: A novel ultra high-throughput 16S rRNA gene amplicon sequencing library preparation method for the Illumina HiSeq platform
Source: Microbiome. 2017 Jul 6;5:68. doi: 10.1186/s40168-017-0279-1 (PMC5501495; doi:10.1186/s40168-017-0279-1)

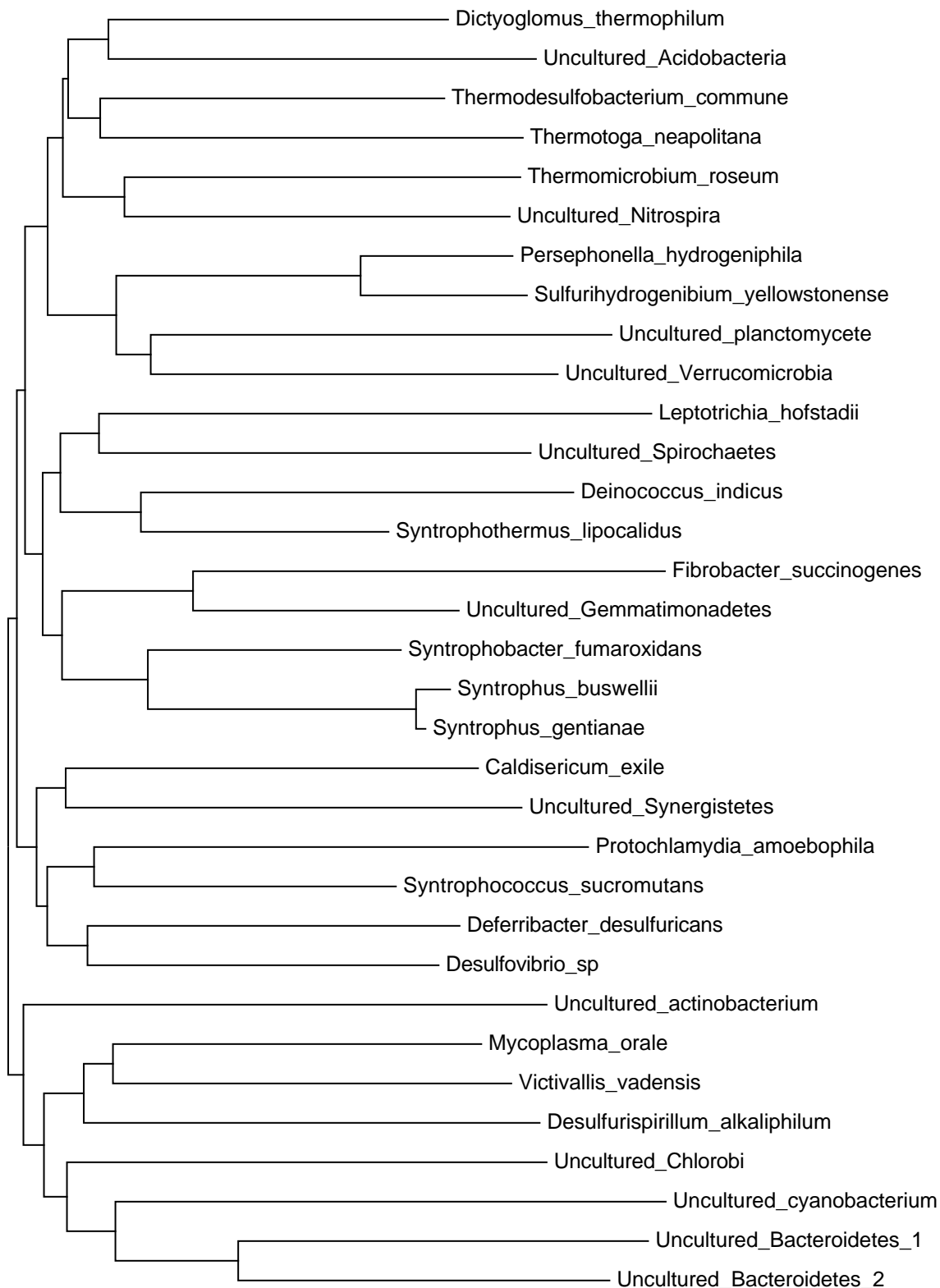

Supplement: Supplementary file 3 — Phylogeny of bacteria in the mock community. Neighbor joining tree showing the phylogenetic relationship among the 33 species represented in the mock community used for method validation. The tree is based on the 515-806 fragment (V4 region) of the 16S rRNA gene. The scale bar at the bottom refers to the number of pairwise nucleotide differences. (PDF 11 kb) [file 40168_2017_279_MOESM3_ESM.pdf]

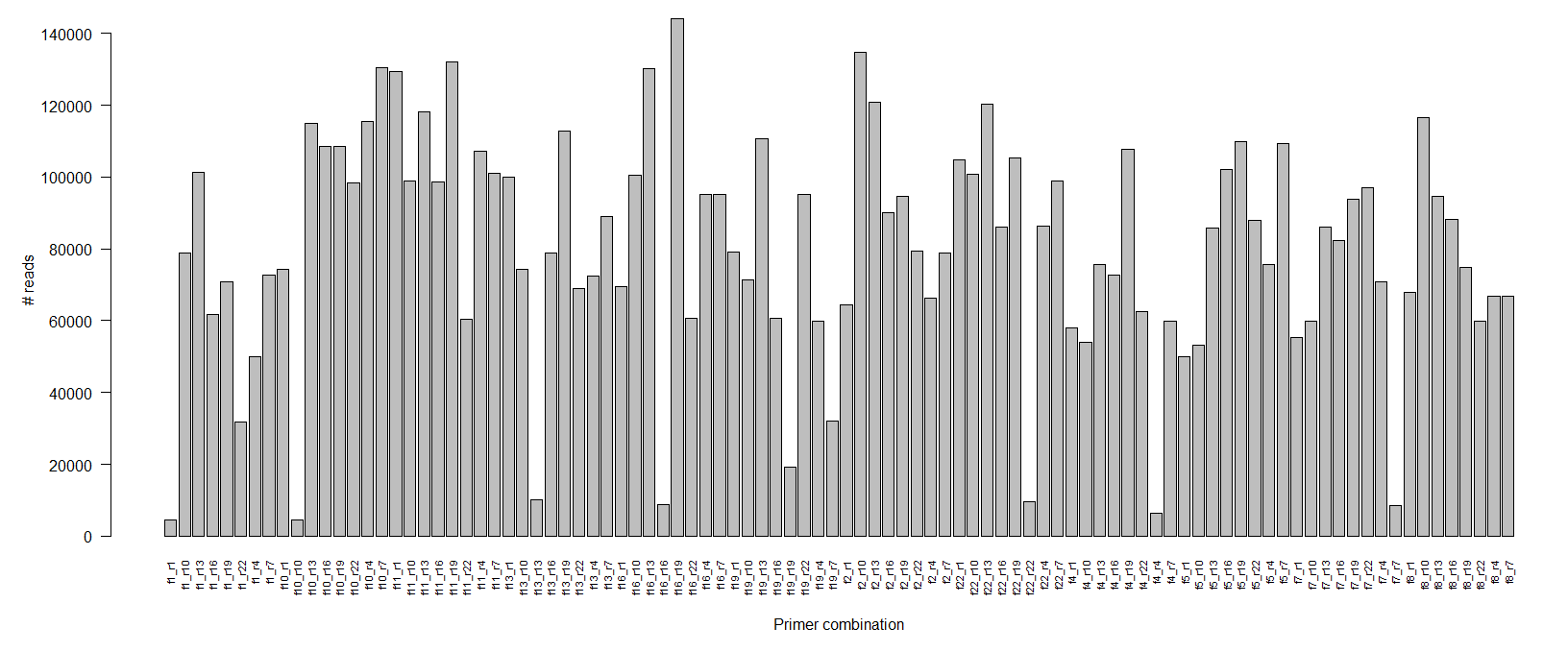

Supplement: Supplementary file 11 — Read counts for all PCR1 primer combinations in Dataset 1. Each bar represents the read count of a mock community sample after quality filtering and paired read merging. The figure shows data for all 96 PCR1 index combinations (Dataset 1, Additional file 4: Table S3). Tenfold fewer reads were observed in samples that were amplified with primer pairs using the same forward and reverse indices. (BMP 1083 kb) [file 40168_2017_279_MOESM11_ESM.bmp]

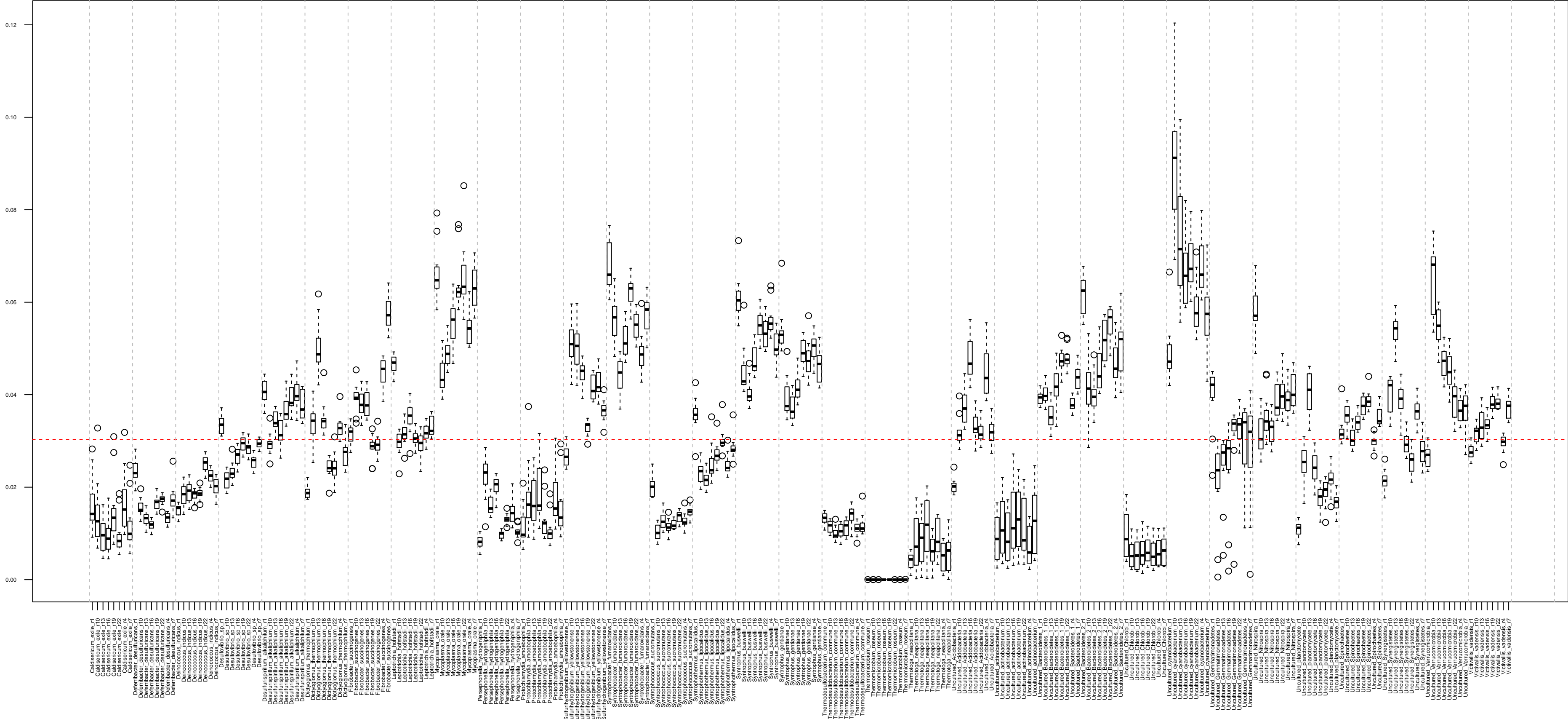

Supplement: Supplementary file 12 — Effects reverse primer indices used for PCR1 on relative abundance estimates in the mock community (Dataset 1, Additional file 4: Table S3). For each species, the eight boxes displayed shows relative abundance estimates obtained using each of the eight indexed PCR1 primers, with the primer name appended to the species names. Each set of measurements results from 12 replicates. Species are separated by grey dotted lines. Each box represents the interquartile range while the whiskers represent 1.5 times the interquartile range. Points outside the whiskers represent outliers. (PDF 27 kb) [file 40168_2017_279_MOESM12_ESM.pdf]

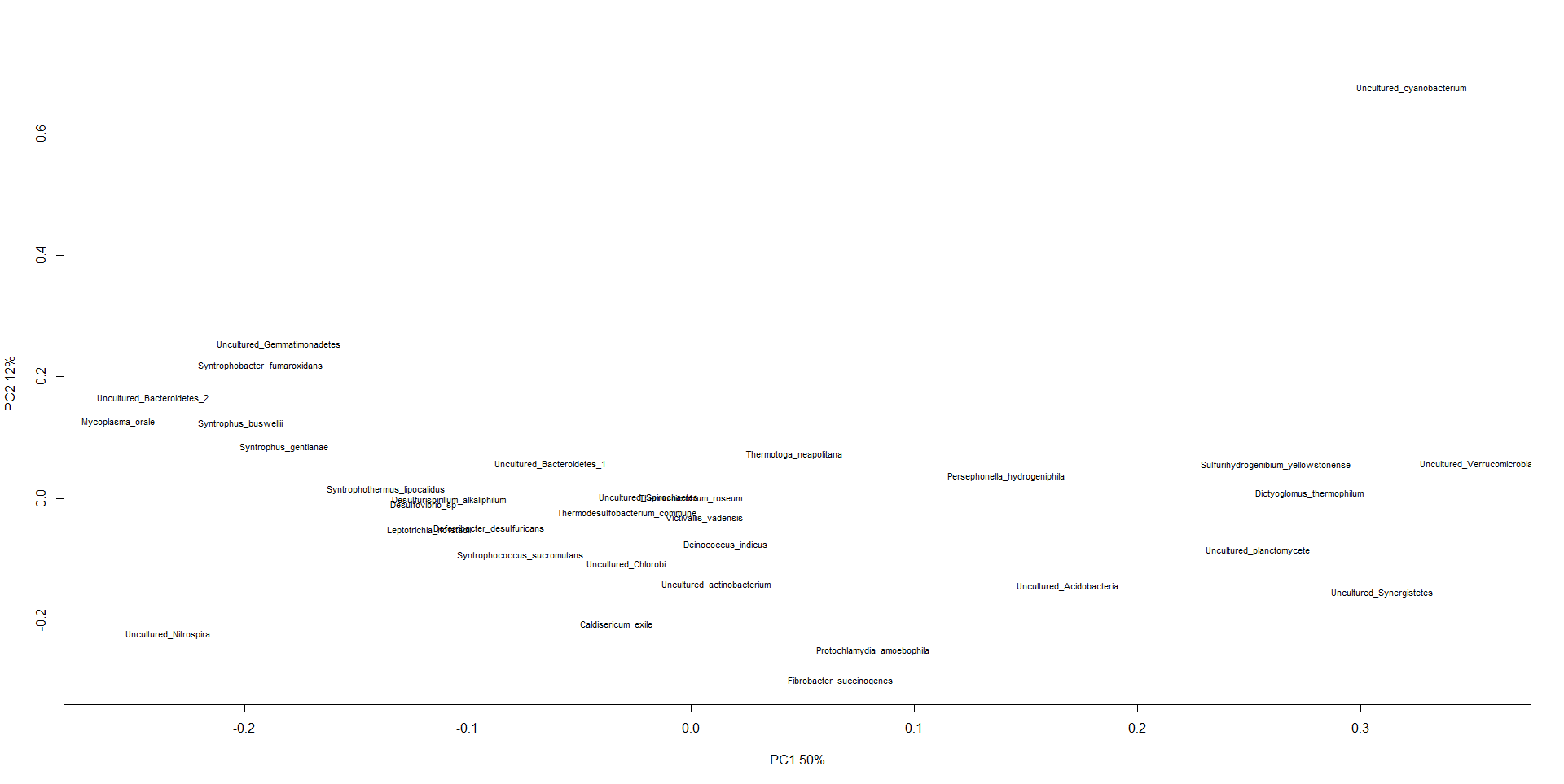

Supplement: Supplementary file 13 — Principal components analysis loadings plot showing the bacterial species defining the two largest variance components in Dataset 1. The main axis of variation accounts for 50% of the total and defines the difference between samples amplified with PCR1 primers r1 (e.g., Mycoplasma orale) and r10 and r13 (e.g., uncultured verrucomicrobium). (BMP 1806 kb) [file 40168_2017_279_MOESM13_ESM.bmp]

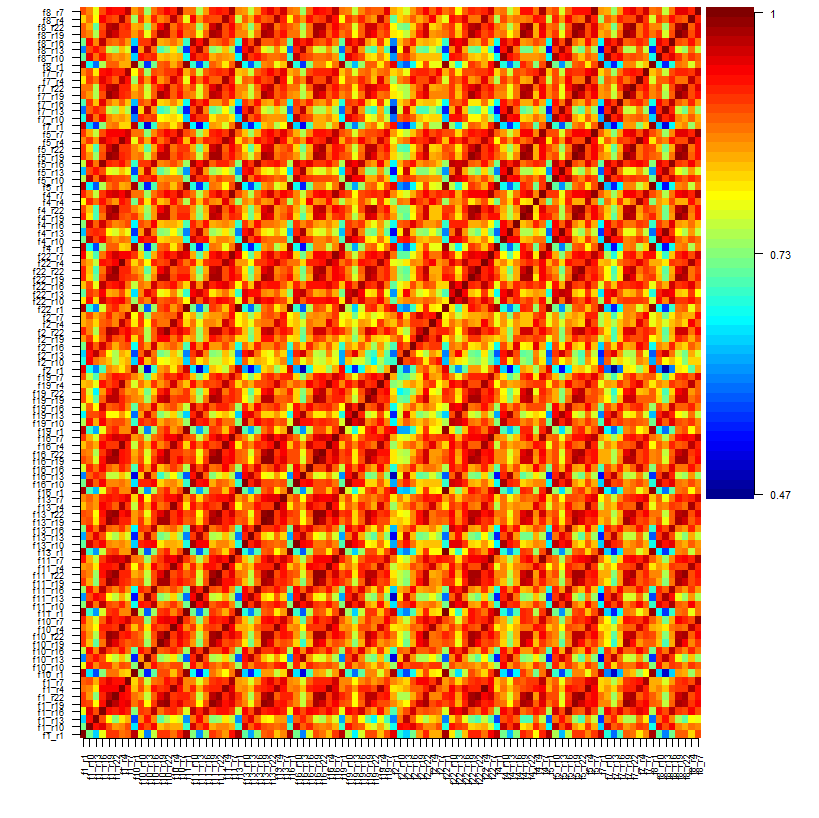

Supplement: Supplementary file 14 — Heat map of pairwise sample correlations in mock community MiSeq data. Pairwise Spearman correlations between the vectors of estimated relative abundances for mock community samples amplified using all 96 PCR1 primer combinations (Dataset 1, Additional file 4: Table S3). Primer pairs are shown on the x- and y-axis. The color of each cell indicates the degree of correlation, according to the color key on the right side of the figure. (BMP 656 kb) [file 40168_2017_279_MOESM14_ESM.bmp]

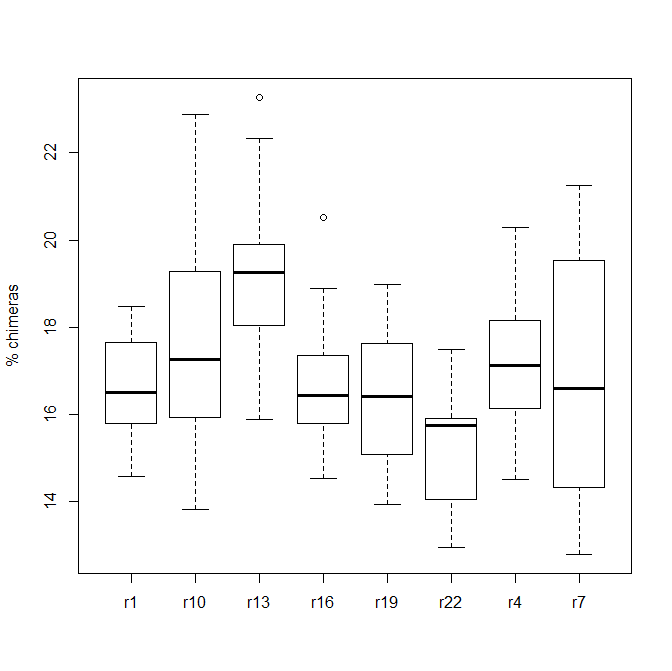

Supplement: Supplementary file 15 — Association between chimeric sequence formation and reverse index sequence from PCR1. The data are the mock community samples amplified using 96 PCR1 primer combinations (dataset 1, Additional file 4: Table S3). Chimera formation was not significantly associated with specific primer combinations, except for a slightly elevated rate for samples amplified with primer r13 (p < 0.001, linear model). Each box represents the interquartile range while the whiskers represent 1.5 times the interquartile range. Points outside the whiskers represent outliers. (BMP 441 kb) [file 40168_2017_279_MOESM15_ESM.bmp]

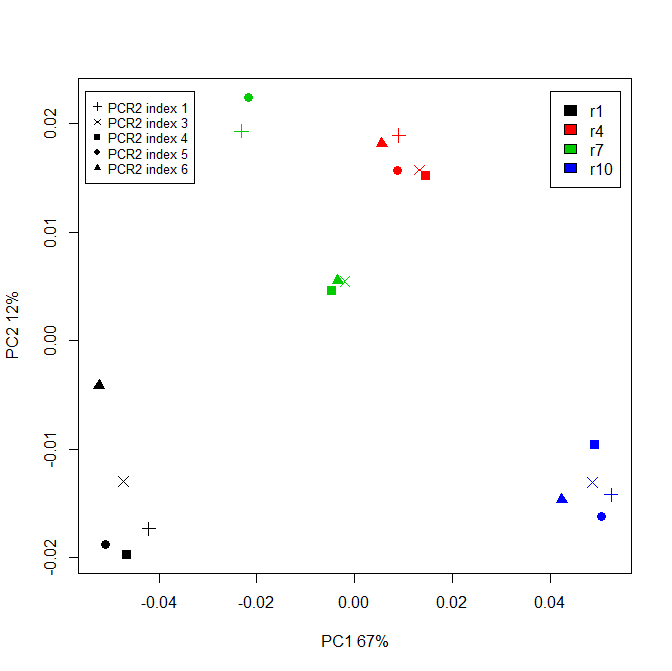

Supplement: Supplementary file 16 — Scores plot based on a principal components analysis model computed from the matrix of species relative abundances in the mock community (dataset 2, Additional file 5: Table S4). Samples are colored according to the reverse primer used for PCR1. Symbol characters represent the different PCR2 reverse primers. The first two dimensions, explaining 79% of the total variance, are shown. (BMP 441 kb) [file 40168_2017_279_MOESM16_ESM.bmp]

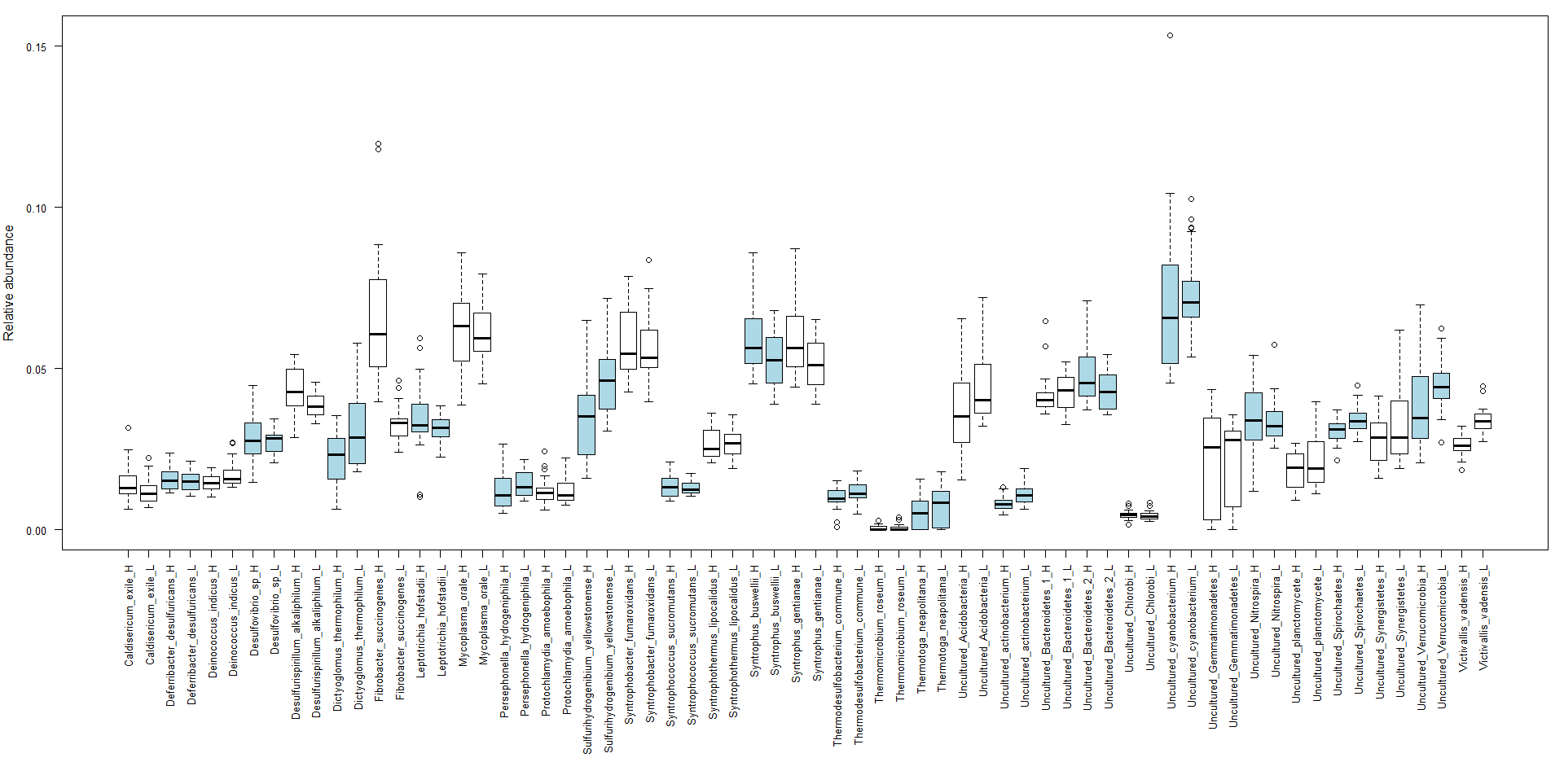

Supplement: Supplementary file 17 — Effects of input DNA amount used for PCR1 on relative abundance estimates in the mock community (dataset 3, Additional file 6: Table S5). For enhanced visualization, each pair of colored bars (alternating blue or white for easier visualization) depicts the estimated relative abundances for one species. Species abundance estimates for high (H) and low (L) input template amounts are shown side-by-side as indicated in the x-axis labels (H = 2.5e7 molecules, L = 2.5e6 molecules). Each set of measurements results from 24 replicates. Each box represents the interquartile range while the whiskers represent 1.5 times the interquartile range. Points outside the whiskers represent outliers. (BMP 1806 kb) [file 40168_2017_279_MOESM17_ESM.bmp]

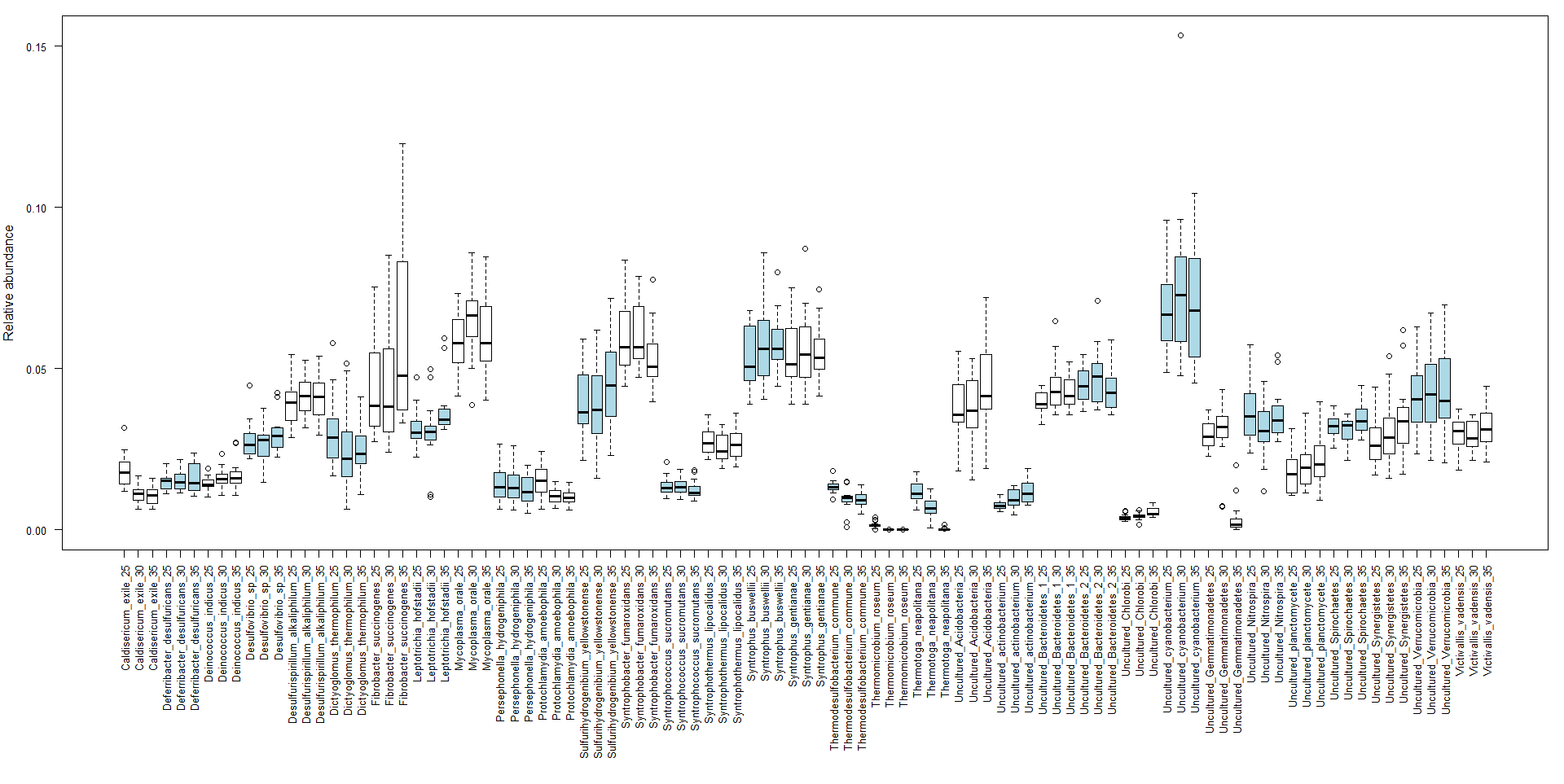

Supplement: Supplementary file 18 — Effects of PCR1 cycle number on relative abundance estimates in the mock community (dataset 3, Additional file 6: Table S5). For enhanced visualization, each alternate triplet of colored bars (blue or white) depicts the estimated relative abundances for one species. Species abundance estimates for 25, 30, and 35 cycles are shown side-by-side as indicated in the x-axis labels. Each set of measurements results from 16 replicates. Each box represents the interquartile range while the whiskers represent 1.5 times the interquartile range. Points outside the whiskers represent outliers. (BMP 1806 kb) [file 40168_2017_279_MOESM18_ESM.bmp]

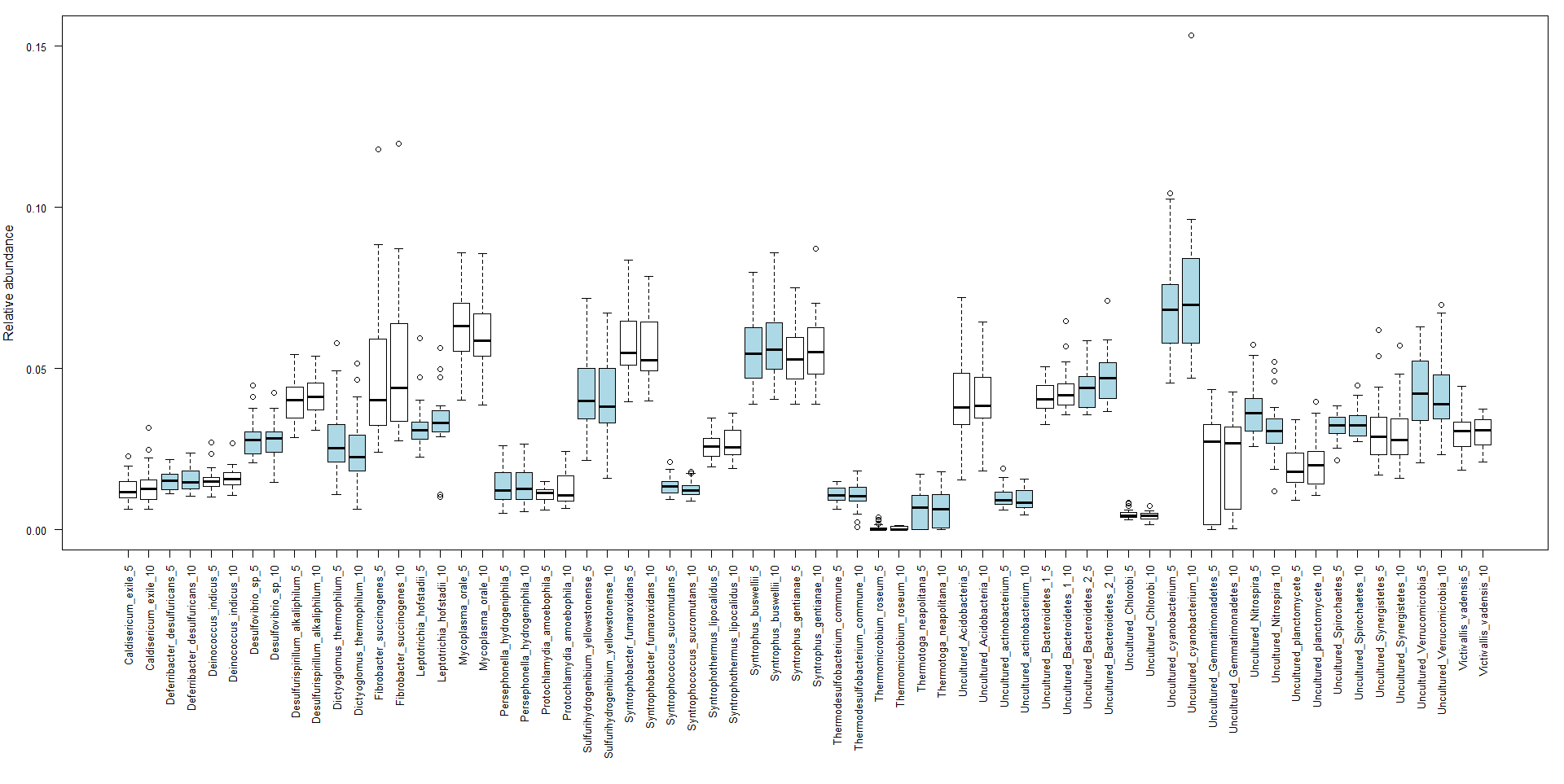

Supplement: Supplementary file 19 — Effects of PCR2 cycle number on relative abundance estimates in the mock community (dataset 3, Additional file 6: Table S5). For enhanced visualization, each alternate pair of colored bars (blue or white) depicts the estimated relative abundances for one species. Species abundance estimates for 5 and 10 cycles are shown side-by-side as indicated in the x-axis labels. Each set of measurements results from 24 replicates. Each box represents the interquartile range while the whiskers represent 1.5 times the interquartile range. Points outside the whiskers represent outliers. (BMP 1806 kb) [file 40168_2017_279_MOESM19_ESM.bmp]

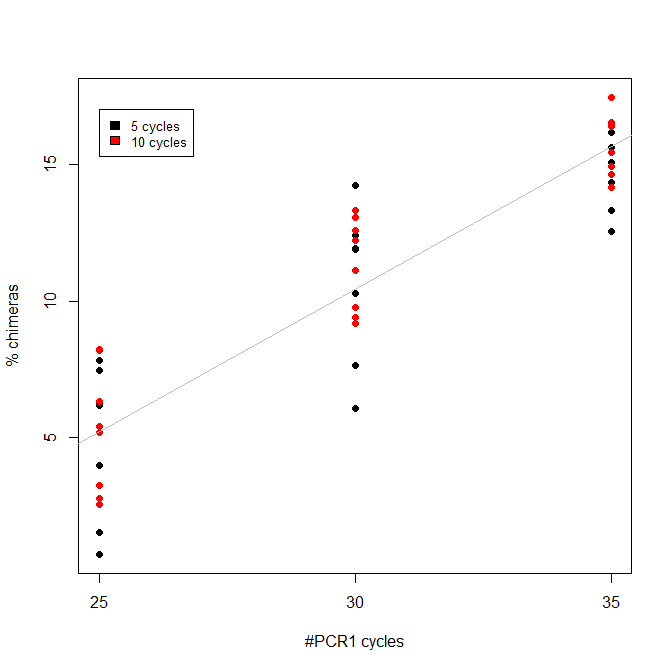

Supplement: Supplementary file 20 — Relationship between PCR1 cycle number and chimeric sequence formation in dataset 3 (Additional file 6: Table S5). The number of PCR1 cycles is indicated on the x-axis. Black and red dots indicate samples amplified using 5 and 10 cycles for PCR2, respectively. A highly significant linear relationship (p < <0.001, linear regression model) was observed. (BMP 441 kb) [file 40168_2017_279_MOESM20_ESM.bmp]

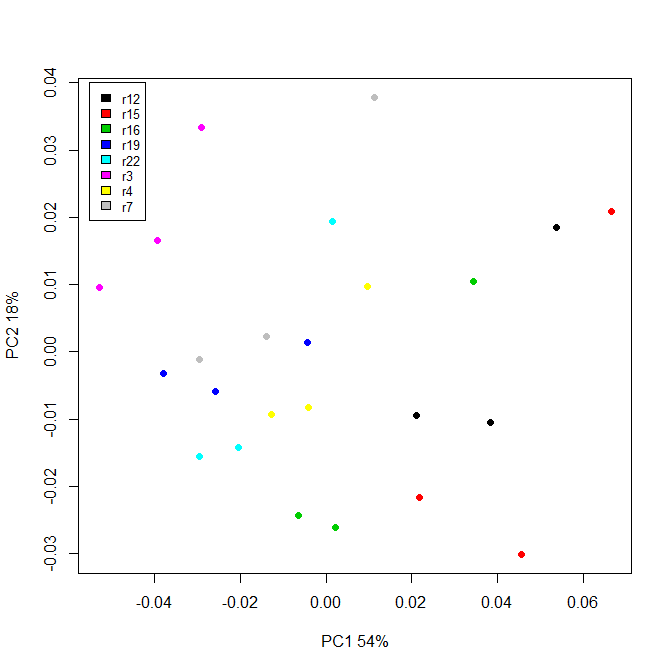

Supplement: Supplementary file 21 — Effects of reverse PCR1 primer indexing in HiSeq data. Scores plot based on a principal component analysis model computed from the matrix of species relative abundances in the mock community sequenced on the HiSeq (dataset 4, Additional file 7: Table S6). Samples are colored according to the reverse primer used for PCR1. The first two dimensions, explaining 72% of the total variance, are shown. (BMP 441 kb) [file 40168_2017_279_MOESM21_ESM.bmp]

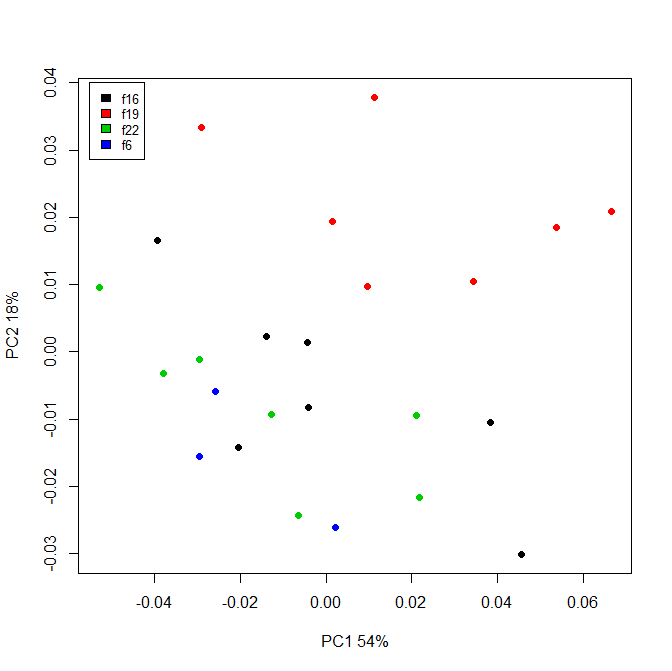

Supplement: Supplementary file 22 — Effects of forward primer indexing in HiSeq data. Scores plot based on a principal component analysis model computed from the matrix of species relative abundances in the mock community sequenced on the HiSeq (dataset 4, Additional file 7: Table S6). Samples are colored according to the forward primer used for PCR1. The first two dimensions, explaining 72% of the total variance, are shown. (BMP 441 kb) [file 40168_2017_279_MOESM22_ESM.bmp]

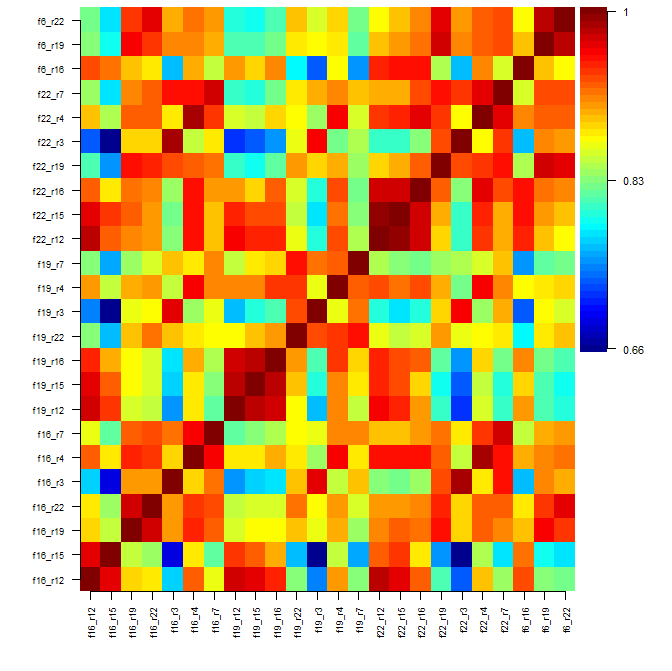

Supplement: Supplementary file 23 — Heat map of pairwise sample correlations in mock community HiSeq data. Pairwise Spearman correlations between the vectors of estimated relative abundances for mock community samples amplified using 24 PCR1 primer combinations and sequenced on the HiSeq (dataset 4, Additional file 7: Table S6). Primer pairs are shown on the x- and y-axis. The color of each cell indicates the degree of correlation, according to the color key on the right side of the figure. (BMP 441 kb) [file 40168_2017_279_MOESM23_ESM.bmp]
